# Controlled-release urea application and optimized nitrogen applied strategy reduced nitrogen leaching and maintained grain yield of paddy fields in Northwest China

**DOI:** 10.3389/fpls.2023.1033506

**Published:** 2023-01-26

**Authors:** Ruliang Liu, Ying Wang, Yu Hong, Fang Wang, Xinping Mao, Jun Yi

**Affiliations:** ^1^ Institute of Agricultural Resources and Environment, Ningxia Academy of Agro-forestry Science, Yinchuan, China; ^2^ National Agricultural Environment Yinchuan Observation and Experiment Station, Ningxia Academy of Agro-forestry Science, Yinchuan, China; ^3^ Hubei Province Key Laboratory for Geographical Process Analysis and Simulation, Central China Normal University, Wuhan, China

**Keywords:** nitrogen leaching, nitrogen uptake, nitrogen use efficiency, nitrogen balance, paddy yield

## Abstract

Nitrogen loss from paddy fields contributes to most of the nitrogen pollution load in the Ningxia Yellow River irrigation area, threatening the water quality of the Yellow River. Consequently, optimizing the nitrogen management practices in this area is essential, which can maintain paddy grain productivity and reduce nitrogen loss simultaneously. Five treatments with different nitrogen application rates and nitrogen fertilizer types were set in this study, including conventional urea application with zero nitrogen application rate (CK, 0 kg hm^-2^), nitrogen expert-based fertilization application strategy (NE, 210 kg hm^-2^), optimized nitrogen fertilizer application strategy recommended by local government (OPT, 240 kg hm^-2^), and farmer’s experience-based nitrogen fertilizer application strategy (FP, 300 kg hm^-2^), and controlled-release urea application (CRU, 180 kg hm^-2^). The data from one growth season field experiment in 2021 revealed the dynamics of nitrogen concentration, paddy yield and its nitrogen uptake characteristic, and nitrogen balance in the paddy field under different nitrogen application practices. Most nitrogen leaching was observed during the seedling and tillering stages in the form of nitrate nitrogen (NO_3_^–^N). Compared with the FP, the CRU and OPT significantly reduced the nitrogen concentrations of total nitrogen (TN), ammonium nitrogen (NH_4_^+^-N), and NO_3_^–^N in the surface and soil water and reduced the nitrogen leaching at 100 cm soil depth. Meanwhile, the paddy grain yield in CRU (7737 kg hm^-2^) and OPT (7379 kg hm^-2^) was not significantly decreased compared with FP (7918 kg hm^-2^), even though the nitrogen uptake by grain and straw was higher in FP (135 kg hm^-2^) than in other treatments (52.10~126.40 kg hm^-2^). However, the grain yield in NE (6972 kg hm^-2^) was decreased compared with the FP. The differences in grain yield among these treatments were mainly attributed to the ear number and grain number changes. Also, the highest nitrogen use efficiency (40.14%), apparent nitrogen efficiency (19.53 kg kg^-1^), and nitrogen partial productivity (43.98 kg kg^-1^) were identified in CRU than in other treatments. Considering increased grain yield and reducing nitrogen loss in the paddy field simultaneously, the treatments of CRU (i.e., 180 kg hm^-2^ nitrogen application rate with controlled-release urea) and OPT (i.e., 240 kg hm^-2^ nitrogen application rate with conventional urea) were recommended for nitrogen fertilizer application in the study area.

## Introduction

Paddy (*Oryza sativa* L.) is one of China’s most important grain crops, producing 37.31% of the total paddy grain yield worldwide (Hua et al., 2019). In order to maintain the high production of paddy grain with limited farmland area, massive nitrogen fertilizer was used in the paddy fields in China, with the nitrogen application rate increasing year by year (Liang et al., 2013; Chen et al., 2014; Shcherbak et al., 2014). It is reported that China consumed 30% of the chemical nitrogen fertilizer around the world, while the nitrogen use efficiency (NUE) in China (25%) was much lower than those in European (52%) and North American countries (Ke et al., 2016). Consequently, plenty of adverse effects appeared when the nitrogen fertilizer was overused, including reduced NUE, increased greenhouse gas emission, accelerated soil acidification, and aggregated water pollution (Kim et al., 2005; Ju et al., 2009; Kakuturu et al., 2013; Liu et al., 2013; Huang et al., 2017). Therefore, many studies were conducted in paddy fields to improve the NUE and reduce the adverse environmental effects aroused by nitrogen fertilizer application without reducing the paddy grain yield at the same time (Shcherbak et al., 2014; Xu et al., 2014a; Zhang et al., 2016; He et al., 2022).

The effects of applied nitrogen fertilizer amount on paddy growth indexes and nitrogen loss characteristics were widely reported (Ju et al., 2009; Zhang et al., 2010; Ruidisch et al., 2013; Chen et al., 2017), as the yield and nitrogen loss amount was directly affected by the fertilizer application rate. Based on the results from previous studies, the optimized nitrogen application amounts in the paddy field in different regions in China were pronounced (Yang et al., 2012; Xu et al., 2014a; Li et al., 2015). In order to increase the NUE and reduce non-productive nitrogen loss further, the effects of nitrogen fertilizer application strategy and fertilizer types on paddy nitrogen uptake and nitrogen loss were also evaluated (Gaudin, 2012; Xu et al., 2014b; Huang et al., 2021). The methods of soil testing and fertilizer recommendation (STFR) and nitrogen expert-based fertilization application strategy (NE) were two of the widely recommended nitrogen application techniques. The STFR technique considers the differences in soil nutrient content, crop nutrient demand characteristics, and fertilizer varieties. Moreover, it provides optimized information on applied fertilizer amount, time, and method to increase fertilizer use efficiency and reduce fertilizer loss (He et al., 2022). As the local government has detailed soil data and is responsible for the optimized fertilizing recommendation, the STFR can also be called an optimized nitrogen fertilizer application recommended by the local government (OPT). Also, the NE is computer software for recommending fertilizer applications (Pampolino et al., 2012). The applied fertilizer amount and time will be recommended by NE when the target yield is set, which also considers the differences in crop varieties, soil properties, and cultivation and management methods (Xu et al., 2014b). In addition, the application of controlled-release urea (CRU) was an exemplary method for reducing nitrogen loss and providing paddy grain yield (Kiran et al., 2010; Sun et al., 2020). The CRU is coated with polymers that slowly release nitrogen for plant uptake (Fageria and Carvalho, 2014; Sun et al., 2022), once applied as base fertilizer and can save labor costs (Cheng et al., 2020). Furthermore, the non-productive nitrogen was reduced while the paddy grain yield and NUE were increased simultaneously (Du et al., 2016; Tian et al., 2019; Sun et al., 2020; Hou et al., 2021). Although these nitrogen management methods (i.e., OPT, NE, and CRU) were positively evaluated in some studies (Yang et al., 2013; Ke et al., 2016; He et al., 2022), the effects of these methods on paddy grain production and nitrogen loss were less compared in the same region during the same study period. Also, the performances of these methods were affected by the soil properties, climate conditions, and paddy specifications. Hence, further studies should be conducted to evaluate these nitrogen management practices on paddy grain production and nitrogen loss.

Ningxia Yellow River irrigation area is located in the arid region of North-west China, one of the commodity grain bases in China (Zhang et al., 2014). The agriculture system strongly depends on the Yellow River, the essential water resource for farmland irrigation in this region (Liu et al., 2019), as the annual precipitation was always no more than 200 mm. Due to the slight soil salinization in this area, the irrigation amount was much higher than the crop water requirement for reducing the soil salt content (Zhang et al., 2014). Consequently, the percolation in the farmland was extremely large and much higher than in other irrigation areas, accelerating dissolved nutrient (e.g., nitrogen, phosphate) percolation. It is reported that 40% of the irrigation water (i.e., three billion m^3^) was percolated and flew back to the Yellow River (Zheng et al., 2018), which threatened the water quality of the local water system and even the Yellow River (Liu et al., 2019; Zhang et al., 2020). Paddy was the main grain crop in the Ningxia Yellow River irrigation area, which was supported by the large irrigation amount (~2000 mm per year) and nitrogen fertilizer input (>300 kg hm^-2^ per year). The vast input of water and nitrogen fertilizer resulted in a low NUE and high nitrogen loss ratio (20%~65%) (Zhang et al., 2010), which resulted in a high nitrogen concentration in both surface water and shallow groundwater (Zhang et al., 2014; Liu et al., 2015). Also, Cao et al. (2011) reported that the paddy field in this area contributed 72% of the nitrogen pollution load in agricultural wastewater. Hence, it is essential to enhance the NUE and reduce the nitrogen loss in the paddy field for non-point source water pollution control in the Ningxia Yellow River irrigation area (Zhang et al., 2020; Zhang et al., 2021). Although many studies evaluated the effects of nitrogen fertilizer application strategy or fertilizer type on grain yield and nitrogen loss in the paddy field, fewer were conducted in the Ningxia Yellow River irrigation area during the same period.

In this study, it is assumed that the performance of nitrogen concentration in both water and soil, nitrogen uptake by paddy plant, and nitrogen loss in paddy fields varied among these positively evaluated nitrogen application treatments (i.e., OPT, NE and CRU). The objectives of this study were to explore the optimized nitrogen treatments for realizing high paddy grain yield and low nitrogen loss amount simultaneously in the Ningxia Yellow River irrigation area.

## Materials and methods

### Study area

The experiment site was located in the field experiment station of Ningxia Academy of Agriculture and Forestry Sciences (106°22′14″E, 38°47′62″N), Ningxia Hui autonomous region, Northwest China. This site belongs to the critical zone of the Ningxia Yellow River irrigation area, characteristic of the middle temperate arid climate zone. The average annual temperature is 8.7 °C, and the cumulative annual sunshine hours are 2867 h. The average annual precipitation is 200 mm, with about 62% of precipitation observed from July to September. At the same time, the annual pan evaporation is 1470 mm, which is much higher than the precipitation. The main crop in this area contains paddy, maize (*Zea mays* L.), and wheat (*Triticum aestivum* L.), frequently irrigated by Yellow River water, ascribing to the limited precipitation. The primary soil type in this area is anthropogenic-alluvial soil with coarse soil texture, characterized by a high sand content (>50%) and low clay content (<5%). Also, the contents of soil organic matter, total nitrogen, and available nitrogen were relatively low. The soil properties are shown in **Table 1**.

**Table 1:** The basic soil properties in the study area.

Depth (cm)	Bulk density (g cm^-3^)	Total porosity (%)	Total salt (g kg^-1^)	Organic matter (g kg^-1^)	Total nitrogen (g kg^-1^)	Available nitrogen (mg kg^-1^)
0-20	1.36	48.7	0.49	10.74	1.01	38.66
20-40	1.36	48.8	0.40	8.71	0.85	26.98
40-60	1.53	42.3	0.39	5.26	0.40	25.12
60-80	1.64	39.0	0.35	4.41	0.31	24.31
80-100	1.44	45.4	0.31	3.15	0.29	23.58

### Experimental details

Five nitrogen application treatments were set in this study, including conventional urea application with zero nitrogen application rate (CK), nitrogen expert-based fertilization application strategy (NE), optimized nitrogen fertilizer application recommended by local government (OPT), and farmer’s experience-based fertilizer application strategy (FP), and controlled-release urea application (CRU). The total nitrogen application amounts in CK, CRU, NE, OPT, and FP were 0, 180, 210, 240, and 300 kg hm^-2^, respectively. At the same time, the same phosphate (P_2_O_5_, 90 kg hm^-2^) and potassium (K_2_O, 45 kg hm^-2^) amounts were used for five treatments.

Three replicated plots were set for each treatment, with an area of 60 m^2^ for each plot. These plots were separated with plastic film from 30 cm above the soil surface to 100 cm soil depth, which can prevent the lateral water flow among these plots. The paddy field was cultivated with the direct-seed method on 4^th^ May 2021, with 20 cm line spacing and 8~10 cm row spacing, respectively. The nitrogen fertilizer used in CRU was polymer-coated urea, while it was conventional urea for NE, OPT, and FP treatments. The polymer-coated urea was once applied as base fertilizer (i.e., on 3^rd^ May) in CRU, while 40%, 25%, and 35% of the conventional urea were applied on 3^rd^ May (base fertilizer), 6^th^ June (during the seeding stage), and 25^th^ June (during the tillering stage) in NE, respectively. Similarly, 50%, 20%, and 30% of the conventional urea were applied one day before sowing, during the seeding stage, and during the tillering stage in both OPT and FP, respectively. Meanwhile, the phosphate and potassium were applied one day before sowing as base fertilizer. During the paddy growth period (i.e., 4^th^ May to 28^th^ September), each plot was irrigated eighteen times with a 2145 mm irrigation amount, and the total precipitation was 98 mm.

### Sampling and laboratory analysis

In the center area of each plot, two water suction cups were installed at 20 and 100 cm soil depth, respectively, which were used to collect soil water samplers. During the paddy growth period, the soil water and field surface samples were collected in 1~15 days intervals, with a shorter time interval after applying nitrogen fertilizer than at other times. After then, these samples were analyzed in the laboratory for the items of total nitrogen (TN) concentration, ammonium nitrogen (NH_4_^+^-N) content, and nitrate nitrogen (NO_3_^–^N) content. Meanwhile, soil samples were collected with a soil auger from the soil surface to 100 cm soil depth in 20 cm intervals before paddy seed sowing (3^rd^ May) and after paddy harvest (28^th^ September), which were used for NH_4_^+^-N and NO_3_^–^N measurements. Also, the irrigation water and precipitation samples were collected, and the concentrations of TN, NH_4_^+^-N, and NO_3_^–^N were determined. During the paddy harvest period, grain and aboveground straw biomass were measured in each plot. Meanwhile, the total nitrogen contents of grain and straw were measured. The total N concentrations in field surface water, soil water, irrigation water, and precipitation were determined by Per sulfate-UV spectrophotometry (Bao, 2000), while the concentrations/contents of NH_4_^+^-N and NO_3_^–^N in these water samples and soil samples were measured by continuous-flow nitrogen analyze (Skalar, San Plus System, Netherlands).

### Calculations

Based on the above data, the NUE (%), nitrogen agronomic efficiency (NAE, kg kg^-1^), nitrogen partial productivity (NPP, kg kg^-1^), and apparent nitrogen loss amount (ANL, kg hm^-2^) were calculated as follows:


(1) $$\text{NUE }\left(\%\right)=\frac{\left(U_{N}-U_{N0}\right)}{F_{N}}\times 100$$

Where *U*_N_ and *U*_N0_ are the total N uptake (kg hm^-2^) at the maturity stage with and without nitrogen fertilizer input, respectively, and *F*_N_ is the amount of nitrogen fertilizer input.


(2) $$\text{NAE}=\frac{GY_{N}}{F_{N}}$$

Where *GY*_N_ is the paddy grain yield (kg hm^-2^) in nitrogen fertilizer application treatments at the maturity period.


(3) $$\text{NPP}=\frac{\left(GY_{N\;}-GY_{N0}\right)}{F_{N}}$$

Where *GY*_N0_ is the paddy grain yield (kg hm^-2^) in CK treatments at the maturity period.


(4) $$\text{ANL}=N_{input}-N_{GY}-N_{SY}-N_{leaching}$$

Where *N*_input_ is the total nitrogen input in the paddy field; *N*_GY_ and *N*_SY_ are the N uptake (kg hm^-2^) in paddy grain and straw at maturity period, respectively; *N*_Leaching_ is the total nitrogen leaching amount (kg hm^-2^) at reference soil depth.

As no runoff event was observed during the study period, the nitrogen balance (kg hm^-2^) in the paddy field was calculated as follows:


(5) $$N_{fer}+N_{irr}+N_{pre}+N_{min}=N_{vol}+N_{den}+N_{GY}+N_{SY}+N_{leaching}+N_{\Delta S}$$

Where *N*_fer_, *N*_irr_, *N*_pre_, and *N*_min_ are the nitrogen inputs from fertilizer, irrigation, precipitation, and mineralization, respectively; *N*_vol_ and *N*_den_ are the nitrogen losses by volatilization and denitrification, respectively; *N*_Δs_ in the nitrogen storage change in the soil profile during the study period. In this study, the summary of *N*_vol_, *N*_den_, and *N*_Leaching_ is defined as apparent nitrogen loss, while the summary of *N*_vol_ and *N*_den_ is defined as undefined nitrogen loss.

### Statistical analysis

The statistical software SPSS 22.0 was used for One-way analysis of variance (ANOVA) with the least significant difference (*LSD*) test at the 5% level.

## Results

### Dynamics of nitrogen concentration of the surface water in paddy fields

**Figure 1:**
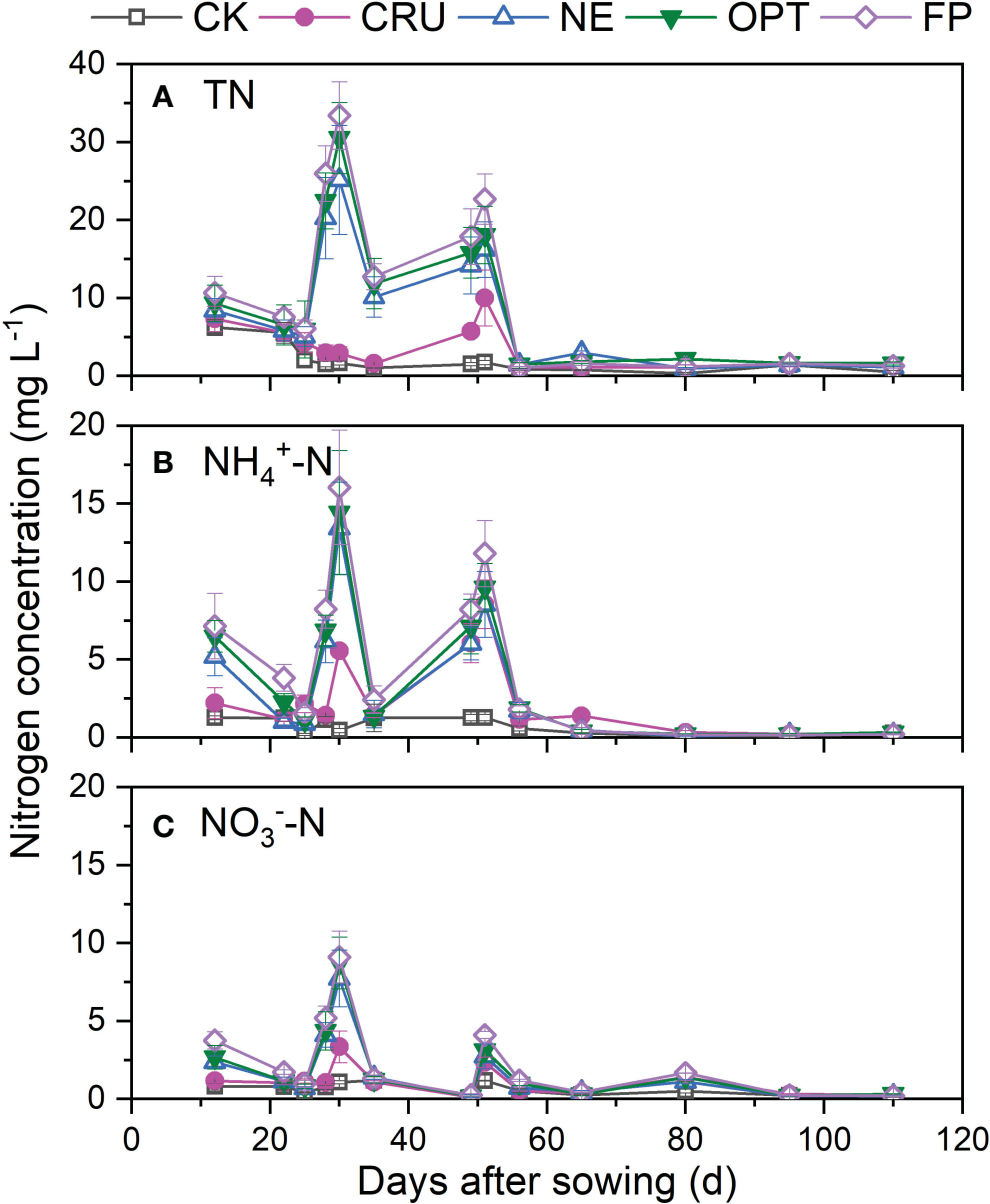
Dynamics of **(A)** TN, **(B)** NH_4_^+^-N, and **(C)** NO_3_^–^N concentration of the surface water in paddy fields under different nitrogen treatments. The vertical bars mean standard deviations of the means CK, zero nitrogen application; CRU, controlled-release urea application; NE, nitrogen expert-based fertilization application strategy; OPT, optimized nitrogen fertilizer application strategy recommended by local government; FP, farmer's experience-based nitrogen fertilizer application strategy.

The nitrogen fertilizer application affected the TN concentration in the surface water, which increased with the nitrogen input amount (**Figure 1A**). Much higher fluctuations in TN concentrations and peak values were identified in NE (25.16 mg L^-1^), OPT (30.55 mg L^-1^), and FP (33.43 mg L^-1^) than in CRU (10.01 mg L^-1^) and CK (6.23 mg L^-1^). For NE, OPT, and FP treatments, the TN concentration reached the highest value on the third day after nitrogen application, then it decreased gradually and reached a shallow level ten days later. In contrast, the relatively high TN concentration (10.33 mg L^-1^) in CRU was only observed on the 52^nd^ day after sowing, nearly 70% lower than that in FP.

The dynamics of NH_4_^+^-N and NO_3_^–^N concentration in the field surface water was similar to the TN concentration (**Figures 1B, C**). Much higher fluctuations in NH_4_^+^-N and NO_3_^–^N concentrations and peak values were identified in NE (13.42 and 7.73 mg L^-1^), OPT (14.44 and 8.73 mg L^-1^), and FP (16.05 and 9.11 mg L^-1^) than in CRU (8.55 and 3.35 mg L^-1^) and CK (1.28 and 1.20 mg L^-1^). For NE, OPT, and FP treatments, the peak value of NH_4_^+^-N and NO_3_^–^N concentrations were about 50% and 20% of the TN concentration, respectively. Also, the peak values of NH_4_^+^-N and NO_3_^–^N concentration were 1~3 days later than that of TN.

### Dynamics of nitrogen concentration of the leaching water at different soil depths in paddy fields

**Figure 2:**
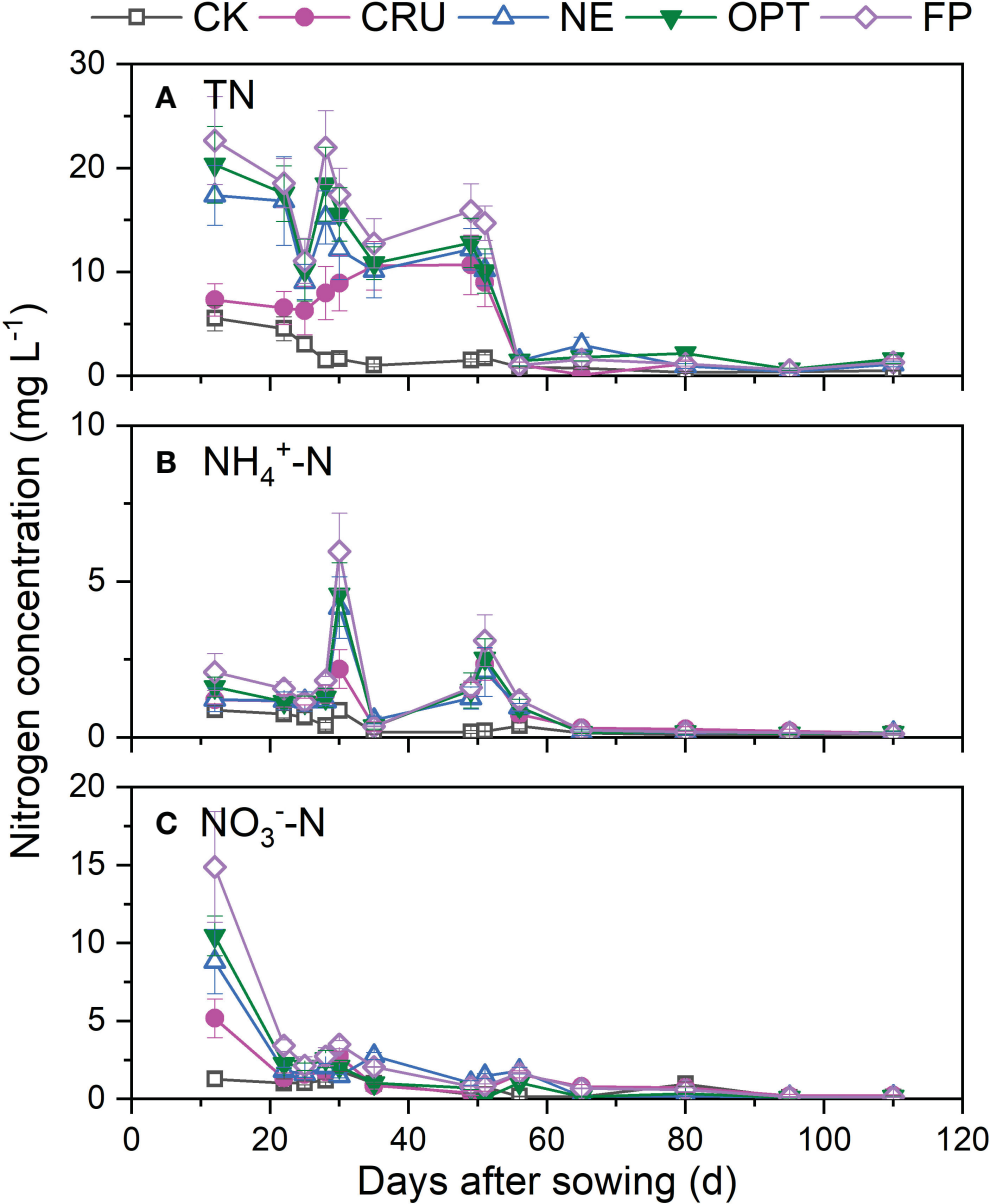
Dynamics of **(A)** TN, **(B)** NH_4_^+^-N, and **(C)** NO_3_^–^N concentration of the leaching water at 20 cm soil depth in the paddy fields under different nitrogen treatments. The vertical bars mean standard deviations of the means CK, zero nitrogen application; CRU, controlled-release urea application; NE, nitrogen expert-based fertilization application strategy; OPT, optimized nitrogen fertilizer application strategy recommended by local government; FP, farmer's experience-based nitrogen fertilizer application strategy.

The TN, NH_4_^+^-N, and NO_3_^–^N concentrations in leaching water at 20 cm soil depth were also dramatically affected by nitrogen fertilizer application (**Figure 2**). The dynamics of TN concentrations between 20 cm soil depth and surface water were similar to some degree. The highest TN concentrations were observed at 12~52 days in CRU (10.70 mg L^-1^), NE (17.39 mg L^-1^), OPT (20.33 mg L^-1^), and FP (22.66 mg L^-1^) after sowing, while the relatively high TN concentrations in CK (5.55 mg L^-1^) were identified at 12~25 days after sowing. After that, the TN concentration was maintained at a low level. The dynamics of NH_4_^+^-N concentration at 20 cm were similar to that of the field surface water, while the peak concentrations at these treatments (0.88~5.97 mg L^-1^) were about 70% lower. Different from the dynamics of TN and NH_4_^+^-N concentrations, the highest NO_3_^–^N concentration was observed on the first day of leaching water sampling (1.80~14.89 mg L^-1^). Then it decreased gradually without an obvious peak value. Similarly, the NE, OPT, and FP treatments had higher concentrations of TN, NH_4_^+^-N, and NO_3_^–^N than CRU and CK at 12~60 days after sowing.

**Figure 3:**
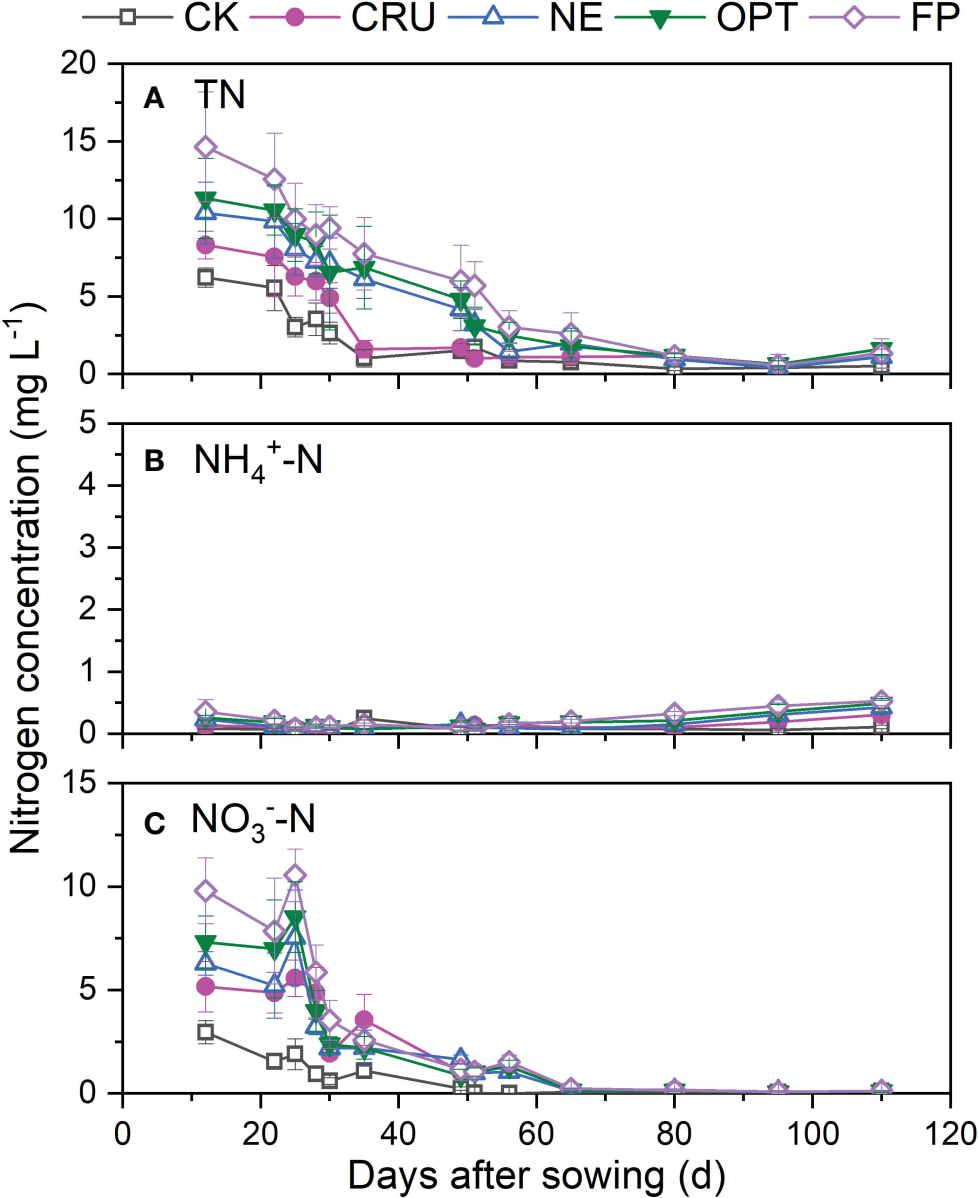
Dynamics of **(A)** TN, **(B)** NH_4_^+^-N, and **(C)** NO_3_^–^N concentration of the leaching water at 100 cm soil depth in the paddy fields under different treatments. The vertical bars mean standard deviations of the means CK, zero nitrogen application; CRU, controlled-release urea application; NE, nitrogen expert-based fertilization application strategy; OPT, optimized nitrogen fertilizer application strategy recommended by local government; FP, farmer's experience-based nitrogen fertilizer application strategy.

**Figure 3** showed that the split fertilizing did not directly affect the dynamics of TN, NH_4_^+^-N, and NO_3_^–^N concentrations in leaching water at 100 cm soil depth. The TN concentration gradually decreased with the paddy growth period extending, which reached a very low value (0~2 mg L^-1^) from 80 days after sowing. From 12 to 50 days after sowing, the TN concentrations were in the order of FP>OPT=NE>CRU>CK. In contrast, no apparent differences were observed after that. The dynamics of NO_3_^–^N concentration were similar to that of TN in 100 cm soil depth. However, the higher concentration in FP (6.82 mg L^-1^) and OPT (4.93 mg L^-1^) than in CRU (2.69 mg L^-1^) was only identified from 12 to 24 days after sowing. Differently, very low NH_4_^+^-N concentrations (<0.7 mg L^-1^) were observed in all treatments, which showed a slow increment during the study period.

### Nitrogen leaching at 100 cm soil depth in paddy fields

Obvious TN leaching was observed in all treatments, while most were identified at the seeding and tillering stages (**Table 2**). It is noticeable that more than 12 kg hm^-2^ of TN was leached in the CK treatment without nitrogen fertilizer application. Meanwhile, significant differences in TN leaching were observed among the five treatments. During the whole paddy growth season, the highest TN leaching was found in FP (59.13 kg hm^-2^) and OPT (48.04 kg hm^-2^), followed by the NE (44.01 kg hm^-2^), with the least TN leaching in CRU (32.91 kg hm^-2^) and CK (12.30 kg hm^-2^). Similarly, a higher TN leaching amount was likely observed in FP, OPT and NE than in CRU and CK in the seeding, tillering, jointing, and booting stages. In comparison, no significant differences in TN leaching amount were identified among all treatments in the filling stage (1.03~2.17 kg hm^-2^).

**Table 2:** Total nitrogen (TN) leaching amount at 100 cm soil depth in paddy fields under different treatments during different growth stages (unit: kg hm^-2^).

Treatments	TN leaching amount at the main growth stages					TN leaching amount at the whole growing season
	SS	TS	JS	BS	FS	
CK	5.88 ± 1.12d	2.63 ± 0.99d	1.95 ± 0.21d	0.81 ± 0.11d	1.03 ± 0.15b	12.30± 2.58d
CRU	18.09 ± 1.99c	8.27 ± 1.11c	2.90 ± 0.25cd	1.67 ± 0.25bc	1.95 ± 0.22a	32.91 ± 3.76c
NE	23.05 ± 1.53bc	12.58 ± 1.55b	4.42 ± 0.35bc	2.16 ± 0.12b	1.80 ± 0.21a	44.01 ± 3.76b
OPT	24.92 ± 2.12b	13.57 ± 0.75ab	5.18 ± 0.75ab	2.2 ± 0.24ab	2.17 ± 0.32a	48.04 ± 4.17ab
FP	31.01 ± 2.91a	15.91 ± 1.31a	7.35 ± 1.68a	2.78 ± 0.32a	2.08 ± 0.29a	59.13 ± 6.51a

SS, seedling stage; TS, tillering stage; JS, jointing stage; BS: booting stage; FS, filling stage; CK, zero nitrogen application; CRU, controlled-release urea application; NE, nitrogen expert-based fertilization application strategy; OPT, optimized nitrogen fertilizer application strategy recommended by local government; FP, farmer’s experience-based nitrogen fertilizer application strategy.

The values before and after the “±” indicate the average value and standard deviation, respectively.

Means within the same column that do not share the same letter are statistically different at P<0.05.

### Residual mineral nitrogen distribution along the soil profile after paddy harvest

**Figure 4:**
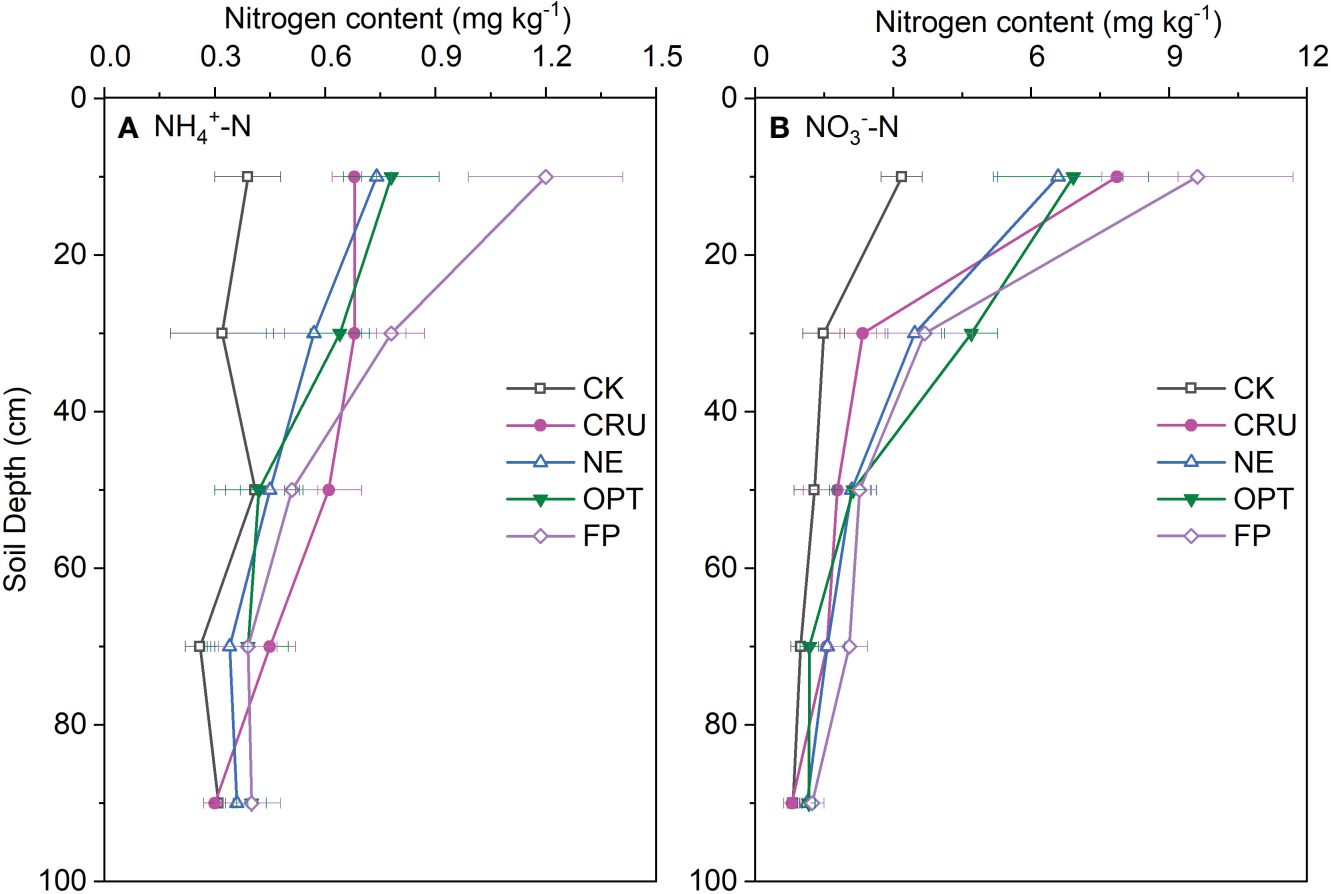
Vertical distribution of **(A)** NH_4_^+^-N and **(B)** NO_3_^–^N contents along the 0-100 cm depth soil profile after paddy was harvested. The horizontal bars mean standard deviations of the means CK, zero nitrogen application; CRU, controlled-release urea application; NE, nitrogen expert-based fertilization application strategy; OPT, optimized nitrogen fertilizer application strategy recommended by local government; FP, farmer's experience-based nitrogen fertilizer application strategy.

**Figure 4** showed that the residual NH_4_^+^-N contents (0.3~1.2 mg kg^-1^) along the soil profile were much lower than the NO_3_^–^N contents (0.8~9.6 mg kg^-1^). With soil depth increasing, both the NH_4_^+^-N and NO_3_^–^N contents decreased gradually. For 0~20 cm soil depths, FP had the highest NH_4_^+^-N and NO_3_^–^N contents (1.20 and 9.62 mg kg^-1^), followed by OPT (0.78 and 6.92 mg kg^-1^), NE (0.74 and 6.59 mg kg^-1^), and CRU (0.68 and 7.87 mg kg^-1^), with the lowest contents in CK (0.39 and 3.19 mg kg^-1^). For 20~40 cm soil depth, the NH_4_^+^-N content in CK (0.32 mg kg^-1^) was significantly lower than other treatments, while the NO_3_^–^N contents were in the order of OPT>FP=NE>CR>CK. However, the five nitrogen treatments identified slight differences in NH_4_^+^-N and NO_3_^–^N contents in the deeper soil layers.

### Grain yield and yield components

**Table 3** showed apparent differences in paddy grain yield and yield components among different nitrogen fertilizer treatments. FP (7918 kg hm^-2^) and CRU (7737 kg hm^-2^) had the highest yield, significantly higher than NE (6972 kg hm^-2^) and CK (4221 kg hm^-2^). In contrast, there was no significant difference in grain yield among FP, CRU, and OPT. Also, higher ear densities were identified in CRU (5.14 million hm^-2^), OPT (5.18 million hm^-2^), and FP (5.13 million hm^-2^) than in CK (3.63 million hm^-2^). At the same time, no significant differences were observed among CRU, NE, OPT and FP or between CK and NE. Similarly, FP (112.33) and CRU (106.50) had a much higher grain spike number than NE (87.27) and CK (70.13). No significant differences in thousand seed weight (24.96~25.39 g) and seed setting rate (84.26%~88.98%) were observed among all treatments.

**Table 3:** The grain yield and yield components under different nitrogen fertilizer treatments after harvest.

Treatment	Grain yield (kg hm^-2^)	Ear density (million hm^-2^)	Grain spike number (-)	Thousand seed weight (g)	Seed setting rate (%)
CK	4221 ± 294c	3.63 ± 0.25b	70.13 ± 6.34c	24.96 ± 0.49a	88.98 ± 3.02a
CRU	7737 ± 261a	5.14 ± 0.31a	106.50 ± 12.30a	25.37 ± 0.54a	84.26 ± 2.05a
NE	6972 ± 462b	4.85 ± 0.78ab	87.27 ± 8.85b	25.39 ± 0.71a	86.65 ± 2.07a
OPT	7379 ± 320ab	5.18 ± 0.635a	99.93 ± 3.97ab	25.27 ± 0.48a	86.12 ± 2.59a
FP	7918 ± 179a	5.13 ± 0.58a	112.33 ± 8.80a	25.22 ± 0.77a	87.77 ± 3.49a

CK, zero nitrogen application; CRU, controlled-release urea application; NE, nitrogen expert-based fertilization application strategy; OPT, optimized nitrogen fertilizer application strategy recommended by local government; FP, farmer’s experience-based nitrogen fertilizer application strategy.

The values before and after the “±” indicate the average value and standard deviation, respectively.

Means within the same column that do not share the same letter are statistically different at P<0.05.

The nitrogen uptake by paddy was strongly affected by the nitrogen application rate, indicated by the much lower nitrogen uptake by grain and straw in CK (34.95 and 17.15 kg hm^-2^) than in other treatments (**Table 4**). Also, the biomass above ground (i.e., grain and straw) in FP (135.14 kg hm^-2^) had more nitrogen amount than NE (114.24 kg hm^-2^), while no significant differences in nitrogen uptake were observed among FP, OPT, and CRU or among OPT, NE, and CRU. Meanwhile, the paddy grain contained the highest nitrogen amount in FP (85.83 kg hm^-2^) and OPT (85.32 kg hm^-2^), followed by CRU (81.27 kg hm^-2^) and NE (76.55 kg hm^-2^), with the lowest amount in CK (34.95 kg hm^-2^). Similarly, the highest *N*_SY_ was observed in FP (49.31 kg hm^-2^) and CRU (43.07 kg hm^-2^). Differently, the NUE was decreased with nitrogen fertilizer application rate, which was in the order of CRU>NE>OPT>FP. In addition, CRU had a much higher MAE and NPP than NE, OPT, and FP, while no significant differences were identified among NE, OPT, and FP.

**Table 4:** Nitrogen uptake, Nutrient use Efficiency (NUE), Nitrogen Agronomic Efficiency (NAE), and Nitrogen Partial Productivity (NPP) under different nitrogen treatments during the paddy growing seasons.

Treatment	N_GY_+N_SY_ (kg hm^-2^)	N_GY_ (kg hm^-2^)	N_SY_ (kg hm^-2^)	NUE (%)	NAE (kg kg^-1^)	NPP (kg kg^-1^)
CK	52.10 ± 0.82c	34.95 ± 2.08c	17.15 ± 1.84c	–	–	–
CRU	124.35 ± 5.62ab	81.27 ± 3.43b	43.07 ± 2.98ab	40.14 ± 2.67a	19.53 ± 1.30a	42.98 ± 1.45a
NE	114.24 ± 5.79b	76.55 ± 6.41b	37.70 ± 0.64b	27.24 ± 2.37c	13.10 ± 1.14b	33.20 ± 2.20b
OPT	126.40 ± 5.05ab	85.32 ± 6.60ab	41.08 ± 2.32b	30.95 ± 1.76b	13.16 ± 0.75b	20.75 ± 1.33b
FP	135.14 ± 15.43a	85.83 ± 13.78a	49.31 ± 5.12a	27.68 ± 4.87c	12.32 ± 2.17b	26.39 ± 0.60b

CK, zero nitrogen application; CRU, controlled-release urea application; NE, nitrogen expert-based fertilization application strategy; OPT, optimized nitrogen fertilizer application strategy recommended by local government; FP, farmer’s experience-based nitrogen fertilizer application strategy; N_GY_: nitrogen uptake by paddy grain; N_SY_: nitrogen uptake by paddy straw.

The values before and after the “±” indicate the average value and standard deviation, respectively.

Means within the same column that do not share the same letter are statistically different at P<0.05.

### Nitrogen balance in 0-100 cm depth soil profile during paddy growth period

The nitrogen balance results (**Table 5**) showed that the nitrogen input from irrigation (precipitation), seed, and mineralization were 25.95, 5.72, and 9.73 kg hm^-2^, respectively. Meanwhile, the nitrogen fertilizer application rate increased the apparent nitrogen loss, nitrogen leaching amount, and undefined nitrogen loss amounts.

**Table 5:** Nitrogen balance in 0-100 cm depth soil profile under different nitrogen fertilizer treatments during the paddy growth period (unit: kg hm^-2^).

Items	CK	CRU	NE	OPT	FP
Total N input	86.27	266.27	296.27	326.27	386.27
Soil mineral nitrogen before transplant	45.23	45.23	45.23	45.23	45.23
Fertilizer	0.00	180.00	210.00	240.00	300.00
Irrigation and precipitation	25.95	25.95	25.95	25.95	25.95
Seed	5.72	5.72	5.72	5.72	5.72
Apparent nitrogen mineralization	9.37	9.37	9.37	9.37	9.37
Total nitrogen output	69.50	166.72	163.90	179.07	197.55
Paddy plant nitrogen uptake	52.10c	124.35ab	114.24b	126.4ab	135.14a
Soil mineral nitrogen after harvest	17.40d	42.37c	49.66b	52.67b	62.41a
Apparent nitrogen loss	16.77d	99.55c	132.37b	147.20b	188.72a
Leaching loss	12.30d	32.91c	44.01b	48.04ab	59.13a
Undefined nitrogen loss	4.47d	66.64c	88.36b	99.16b	129.59a

CK, zero nitrogen application; CRU, controlled-release urea application; NE, nitrogen expert-based fertilization application strategy; OPT, optimized nitrogen fertilizer application strategy recommended by local government; FP, farmer’s experience-based nitrogen fertilizer application strategy.

Apparent nitrogen loss (kg hm^-2^) = nitrogen input (initial soil mineral nitrogen) in the 0-100 cm soil layer + nitrogen fertilizer rate + nitrogen from irrigation and precipitation+ nitrogen in the seed + the apparent Nitrogen mineralization) – nitrogen output (residual soil mineral nitrogen in the 0-100 cm soil layer + paddy plant nitrogen uptake).

The values before and after the “±” indicate the average value and standard deviation, respectively.

Means within the same row that do not share the same letter are statistically different at P<0.05.

## Discussion

### Effects of nitrogen management on nitrogen dynamics and leaching in paddy field

The nitrogen concentration and nitrogen loss in the paddy field were affected by nitrogen fertilizer application. The highest NH_4_^+^-N concentration of surface water was observed 1~3 days after fertilizer application, while the peak value of NO_3_^–^N concentration was 1~3 days after that, which was attributed to the reaction time needed for hydrolysis and nitrification (Wang et al., 2019). Compared with the surface water, the NH_4_^+^-N concentration in the leaching water was much lower than that, especially at 100 cm soil depth (**Figure 3**). The much lowered NH_4_^+^-N concentration in the leached water can be explained by the soil absorption of NH_4_^+^-N (Zhang et al., 2018). Consequently, the NO_3_^–^N was the primary form of nitrogen leaching. Meanwhile, the meager NH_4_^+^-N contents were observed along the soil profile when the paddy was harvested, and they can be explained by thoughtful nitrification as the time for the last fertilizer application was two months ago. This finding was also identified by Yang et al. (2012), who studied the process of nitrogen migration and transformation in paddy fields with controlled-release nitrogen fertilizers. Additionally, it was found that most of the nitrogen leaching happened forty days after seed sowing. This result was attributed to the fertilizer application time distribution. Hua et al. (2019) found that nitrogen leaching mainly occurred in the first fifty days of paddy planting (during fertilization). Hence, the nitrogen fertilizer application rate and time can be improved further, especially considering the high base fertilizer application rate (Zhang et al., 2017). It was found that the net nitrogen leaching amount was in the range of 12.60~38.83 kg hm^-2^, which was much higher than the results from Ke et al. (2016) (1.80~6.65 kg N hm^-2^). This difference was mainly attributed to the sandier soil texture and much large irrigation amount in this study (Witheetriron et al., 2011).

Much higher nitrogen concentration and nitrogen leaching were identified in FP than in other treatments, implying more water pollution by FP treatment. It was reported that optimizing the nitrogen fertilizer application rate was a practical and cost-effective method for controlling nitrogen loss at the source (Kiran et al., 2010; Ruidisch et al., 2013; Zhang et al., 2018). Compared with FP, the much lower nitrogen concentration in the surface and soil water were observed in the treatments with reduced nitrogen fertilizer application (**Figures 1**–**3**), implying less risk for nitrogen leaching. The calculated nitrogen loss amount also confirmed this under different treatments in this study (**Table 2**). Zhang et al. (2018) also found that optimizing agronomic management practices and intercepting nitrogen migration could reduce nitrogen losses by 15% to 82%. Hence, it is better to apply nitrogen fertilizer as little as possible to reduce nitrogen loss. Besides reducing the nitrogen fertilizer application rate, using controlled-released nitrogen fertilizer was another excellent choice for reducing nitrogen loss. The much lower nitrogen concentration indicated this in surface and soil water without apparent peak values (**Figures 1**–**3**) and the lower nitrogen leaching amount compared with FP, OPT, and NE. These observations were consistent with the results of other studies (Wang et al., 2019; Sun et al., 2022), which explained that the nitrogen released rate was slowed down, and the soil absorbed the nitrogen well. In the end, CRU was an excellent choice for nitrogen loss reduction.

### Effects of nitrogen management on paddy plant nitrogen uptake and productivity

Nitrogen is vital for crop production, as the proper nitrogen application rate can promote paddy plant growth and its dry matter accumulation and increase grain yield in the end (Chen et al., 2014). However, the grain yield will not infinitely increase with the nitrogen input amount, which will even decrease when too much nitrogen fertilizer is applied (Zhang et al., 2014). It was found that there was great potential in reducing the nitrogen application rate in the study area, which was indicated by the same grain yield in OPT with 240 kg hm^-2^ nitrogen input compared with FP with 300 kg hm^-2^ nitrogen fertilizer. Furthermore, this was also proved by the findings of Zhang et al. (2014) in the same study area. It showed that those four yield components were not decreased in OPT compared with FP (**Table 2**), while the ear density and grain yield significantly reduced when the nitrogen fertilizer application rate reduced further. However, the yield components of thousand seed weight and seed setting rate were not related to the nitrogen management practices in this study. These findings were also identified by Gen et al. (2015), who pronounced that the thousand seed weight was only affected by the paddy species but not the nitrogen fertilizer application rate. Hence, increasing the ear density and grain spike was crucial for enhancing the grain yield in the paddy field.

However, there is a lower limit for nitrogen fertilizer application, as the grain yield in NE with 210 kg hm^-2^ nitrogen application rate was significantly lower than FP, even though the nitrogen application time was optimized. These results were also supported by the findings of Liu et al. (2015), who found that the paddy grain yield decreased when the nitrogen application rate was reduced by 30% (from 300 kg hm^-2^ to 210 kg hm^-2^) in the same study area. Hence, when the conventional urea was applied, the optimized nitrogen application rate probably ranged from 210 to 240 kg hm^-2^. This optimized fertilizer rate was much higher than the results of Qiao et al. (2013) (150~210 kg hm^-2^), mainly ascribing to the differences in soil nutrient content and texture. The soil parent in this area was mainly from the Yellow River alluvium, which was characterized by low nutrients (e.g., total nitrogen was about 0.8~1.1g kg^-1^) and coarse soil particles (Cao et al., 2011), which limited the soil nutrient supply and increased the nitrogen fertilizer leaching consequently. Hence, it is necessary to increase the soil nutrient contents (e.g., organic matter, total nitrogen) by applying organic fertilizer application and straw incorporation practices (Zhang et al., 2014), reducing the nitrogen fertilizer application rate further.

Compared with the conventional urea fertilizer, the CRU presented a higher potential for fertilizer application rate reduction (Fageria and Carvalho, 2014; Hou et al., 2016). This result was supported by the same grain yield in CRU and OPT, although the total nitrogen input in the CRU was reduced by 40% (180 kg hm^-2^) compared with the FP. This phenomenon was also proved by Gen et al. (2015), who found that the crop yield was not decreased when the total nitrogen was reduced by 50% when the controlled-release urea was used. The reason for the great potential in nitrogen reduction with controlled-release urea was revealed by Liu et al. (2019) and Yang et al. (2012), who pronounced that the nitrogen release rate in CRU was well matched with the nitrogen requirement of the paddy plant. Consistent with Yang et al. (2012) and Li et al. (2017), the NUE in CRU was obviously improved compared with the FP treatment in this study. Also, Husain et al. (2019) found that the CRU treatment (nitrogen fertilizer reduced by 20%) increased grain yield and reduced nitrogen loss from runoff and leaching compared with the FP. These results proved the great potential in total nitrogen reduction with CRU.

In the end, both CRU and OPT treatments maintained paddy grain yield and simultaneously reduced nitrogen loss in the study area. In contrast, the NE and PF had a lousy performance in maintaining grain yield and reducing nitrogen loss, respectively. The optimized total nitrogen application rate was 210~240 kg hm^-2^ and<180 kg hm^-2^ for conventional urea and controlled-release urea, respectively. Noticeably, overmuch irrigation water was consumed in the study area, much higher than the paddy evapotranspiration. As a result, a massive amount of the irrigated water was percolated, which promoted nitrogen leaching further. Hence, water-saving practices should be conducted in the paddy field, which can reduce nitrogen leaching and increase the NUE simultaneously, and the nitrogen application rate can be reduced further as a consequence. Meanwhile, reducing the unit price of controlled-release urea is better, which will promote its popularization and application in the paddy field.

### Shortage and prospect

Indeed, the differences in nitrogen leaching amount and grain yield among these five treatments in this study will vary with paddy cultivation year and climate conditions. Hence, it is better to conduct these experiments for longer, which can help us obtain a more reliable result for choosing an optimized nitrogen application treatment. Meanwhile, the nitrogen loss amount may increase in global warming as more irrigation water will be applied due to the increased evapotranspiration. Meanwhile, the higher temperature resulting from global warming may deteriorate water pollution as the eutrophication process will be accelerated. In addition, the practices of deep placement of nitrogen fertilizer, applying nitrification inhibitor, and optimizing the coat of controlled-release urea fertilizer can reduce nitrogen loss and increase the grain yield further (Kiran et al., 2010; Gaudin, 2012), which have to be evaluated in future in the study area. Also, monitoring NH_3_ volatilization and N_2_O emission under different nitrogen management practices will be done in the future, as these gases have adverse effects on the environment (Katata et al., 2013; Shcherbak et al., 2014) and should be evaluated for optimizing nitrogen application practices.

## Conclusion

This study investigated the dynamics of nitrogen concentration, paddy yield and its nitrogen uptake characteristic, and nitrogen balance in the paddy field under different nitrogen application practices in Northwest China. Compared with the FP, the CRU and OPT significantly reduced the nitrogen concentrations (i.e., TN, NH_4_^+^-N, NO_3_^–^N, and TN) in both surface water and soil water and reduced the nitrogen leaching at 100 cm soil depth. Meanwhile, the grain yield in CRU and OPT was not significantly decreased compared with FP, even though the nitrogen uptake by grain and straw was higher in FP than in other treatments. However, the grain yield in NE was decreased compared with the FP. The differences in grain yield among these treatments were mainly attributed to the ear number and grain number changes. Also, the highest NUE, NAE, and NPP were identified in CRU than in other treatments. Considering increasing grain yield and reducing nitrogen loss simultaneously, the treatments of CRU and OPT were first recommended for nitrogen fertilizer application in the study area. The monitoring of volatilization and denitrification will be done in the future to comprehensively evaluate the adverse environmental effects of nitrogen fertilizer application.

## Data availability statement

The raw data supporting the conclusions of this article will be made available by the authors, without undue reservation.

## Author contributions

RL: writing review and editing, supervision, and project administration. YW: formal analysis, data curation, and investigation. YH: data curation and investigation. FW: sample analysis and investigation. XM: sample analysis and investigation. JY: analyzed the data and led the writing of the manuscript. All authors: contributed to the article and approved the submitted version.

## References

[B1] Bao S. D. (2000). Soil and agro-chemistry analysis, third ed (Beijing: China Agricultural Press).

[B2] Cao Y. C. Feng Y. Z. Yang Y. L. Yang G. H. (2011). GIS analysis of structural characteristics of pollution sources in irrigable farmland in ningxia China. Acta Ecologica Sinica. 31 (12), 3468–3477. doi: 10.3724/SP.J.1011.2011.00468

[B3] Chen J. N. Cao F. B. Xiong H. R. Huang M. Zou Y. B. Xiong Y. F. (2017). Effects of single basal application of coated compound fertilizer on yield and nitrogen use efficiency in double-cropped rice. Crop J. 5 (3), 265–270. doi: 10.1016/j.cj.2017.01.002

[B4] Chen X. P. Cui Z. L. Fan M. S. Vitousek P. Zhao M. Ma W. Q.. (2014). Producing more grain with lower environmental costs. Nature 514, 486–489. doi: 10.1038/nature13609 25186728

[B5] Cheng S. Li S. P. Tian J. Y. Xing Z. P. Hu Y. J. Guo B. W.. (2020). Effects of one-time nitrogen basal application on the yield and quality of different direct-seeding rice crops by machine. Trans. Chin. Soc. Agric. Engineering. 36 (24), 1–10. doi: 10.11975/j.issn.1002-6819.2020.24.001

[B6] Du J. Sun K. G. Lei L. J. He A. L. Zhang Y. H. Sun K. Z. (2016). Effects of combined application of controlled release urea and common urea on activities of key enzymes related with nitrogen metabolism, yield and quality of rice. J. Henan Agric. Sci. 45 (3), 67–72. doi: 10.15933/j.cnki.1004-3268.2016.03.013

[B7] Fageria N. K. Carvalho M. C. S. (2014). Comparison of conventional and polymer coated urea as nitrogen sources for lowland rice production. J. Plant Nutrient. 37 (8), 1358–1371. doi: 10.1080/01904167.2014.888736

[B8] Gaudin R. (2012). The kinetics of ammonia disappearance from deep-placed urea supergranules (USG) in transplanted rice: the effects of split USG application and PK fertiliser. Paddy Water Environment. 10, 1–5. doi: 10.1007/s10333-011-0249-3

[B9] Gen B. Sun Y. B. Zhang M. Li C. L. Yang Y. C. Liu Z. G. (2015). Long-term effects of controlled release urea application on crop yields and soil fertility under rice-oilseed rape rotation system. Field Crops Res. 184, 65–73. doi: 10.1016/j.fcr.2015.09.003

[B10] He P. Xu X. P. Zhou W. Smith W. He W. T. Grant B.. (2022). Ensuring future agricultural sustainability in China utilizing an observationally validated nutrient recommendation approach. Eur. J. Agronomy. 132, 126409. doi: 10.1016/J.EJA.2021.126409

[B11] Hou H. Q. Huang Y. L. Ji J. H. Liu Y. R. Liu X. M. Hu Z. P. (2016). Effects of controlled-release fertilizer application on double cropping rice yield and nitrogen use efficiency. Chin. J. Rice Science. 30 (4), 389–396. doi: 10.16819/j.1001-7216.2016.5163

[B12] Hou P. F. Jiang Y. Yan L. Petropoulos E. Wang J. Y. Xue L. H.. (2021). Effect of fertilization on nitrogen losses through surface runoffs in Chinese farmlands: A meta-analysis. Sci. Total Environment. 793, 148554. doi: 10.1016/J.SCITOTENV.2021.148554 34171810

[B13] Huang S. H. He P. Jia L. L. Ding W. C. Ullah S. Zhao R. G.. (2021). Improving nitrogen use efficiency and reducing environmental cost with long-term nutrient expert management in a summer maize-winter wheat rotation system. Soil Tillage Res. 213, 105117. doi: 10.1016/J.STILL.2021.105117

[B14] Huang T. Ju X. T. Yang H. (2017). Nitrate leaching in a winter wheat-summer maize rotation on a calcareous soil as affected by nitrogen and straw management. Sci. Rep. 7, 42247. doi: 10.1038/srep42247 28176865 PMC5296732

[B15] Hua L. L. Zhai L. M. Liu J. Guo S. F. Li W. C. Zhang F. L.. (2019). Characteristics of nitrogen losses from a paddy irrigation-drainage unit system. Agriculture Ecosyst. Environment. 285 (1), 106629. doi: 10.1016/j.agee.2019.106629

[B16] Husain A. Muneer M. A. Wu F. Yin G. F. Shen S. Z. Wang F.. (2019). Application of optimum n through different fertilizers alleviate NH_4_ ^+^-n, NO_3_ ^–^N and total nitrogen losses in the surface runoff and leached water and improve nitrogen use efficiency of rice crop in erhai lake basin, China. Commun. Soil Sci. Plant Anal. 50, 716–738. doi: 10.1080/00103624.2019.1589482

[B17] Ju X. T. Xing G. X. Chen X. P. Zhang S. L. Zhang L. J. Liu X. J.. (2009). Reducing environmental risk by improving n management in intensive Chinese agricultural systems. Proc. Natl. Acad. Sci. United States America. 106, 3041–3046. doi: 10.1073/pnas.0813417106 PMC2644255 19223587

[B18] Kakuturu S. Chopra M. Hardin M. Wanielista M. (2013). Total nitrogen losses from fertilized turfs on simulated highway slopes in Florida. J. Environ. Engineering. 139 (6), 829–837. doi: 10.1061/(ASCE)EE.1943-7870.0000690

[B19] Katata G. Hayashi K. Ono K. Nagai H. Miyata A. Mano M. (2013). Coupling atmospheric ammonia exchange process over a rice paddy field with a multi-layer atmosphere-soil-vegetation model. Agric. For. Meteorology. 180, 1–21. doi: 10.1016/j.agrformet.2013.05.001

[B20] Ke J. Xing X. M. Li G. H. Ding Y. F. Dou F. G. Wang S. H.. (2016). Effects of different controlled-release nitrogen fertilisers on ammonia volatilisation, nitrogen use efficiency and yield of blanket-seedling machine-transplanted rice. Field Crops Res. 205, 147–156. doi: 10.1016/j.fcr.2016.12.027

[B21] Kim J. S. Oh S. Y. Oh K. Y. (2005). Nutrient runoff from a Korean rice paddy watershed during multiple storm events in the growing season. J. Hydrology 327 (1/2), 128–139. doi: 10.1016/j.jhydrol.2005.11.062

[B22] Kiran J. K. Khanif Y. M. Amminuddin H. Anuar A. R. (2010). Effffects of controlled release urea on the yield and nitrogen nutrition of flooded rice. Commun. Soil Sci. Plant Analysis. 41, 811–819. doi: 10.1080/00103621003592333

[B23] Liang B. Wei Z. Yang X. Y. Zhou J. B. (2013). Fate of nitrogen-15 as influenced by soil and nutrient management history in a 19-year wheat–maize experiment. Field Crops Res. 144, 126–134. doi: 10.1016/j.fcr.2012.12.007

[B24] Li P. F. Lu J. W. Hou W. F. Pan Y. H. Wang Y. Khan M. R.. (2017). Reducing nitrogen losses through ammonia volatilization and surface runoff to improve apparent nitrogen recovery of double cropping of late rice using controlled release urea. Environ. Sci. pollut. Res. 24 (12), 11722–11733. doi: 10.1007/s11356-017-8825-8 28332086

[B25] Liu R. L. Wang F. Zhang A. P. Li Y. H. Hong Y. Yang S. Q.. (2019). Effects of application of controlled-release nitrogen fertilizer on nitrogen use efficiency and leaching of rice in ningxia yellow river irrigation area. Bull. Soil Water Conserv. 33, 251–256. doi: 10.13870/j.cnki.stbcxb.2019.05.037

[B26] Liu X. J. Zhang Y. Han W. X. Tang A. Shen J. Cui Z.. (2013). Enhanced nitrogen deposition over China. Nature 494, 459–462. doi: 10.1038/nature11917 23426264

[B27] Liu R. L. Zhang A. P. Li Y. H. Wang F. Zhao T. C. Chen C. (2015). Rice yield, nitrogen use efficiency (NUE) and nitrogen leaching losses as affected by long-term combined applications of manure and chemical fertilizers in yellow river irrigated region of ningxia, China. J. Agro-Environment Science. 34 (5), 947–954. doi: 10.11654/jaes.2015.05.018

[B28] Li X. Xie G. X. Liu Q. Rong X. M. Yi J. Xie Y.. (2015). Effect of reducing amount of controlled release urea on nitrogen runoff and leakage loss in paddy field. J. Soil Water Conserv. 29 (5), 70–74. doi: 10.13870/j.cnki.stbcxb.2015.05.014

[B29] Pampolino M. F. Witt C. Pasuquin J. M. Johnston A. Fisher M. J. (2012). Development approach and evaluation of the nutrient expert software for nutrient management in cereal crops. Comput. Electron. Agriculture. 88, 103–110. doi: 10.1016/j.compag.2012.07.007

[B30] Qiao J. Yang L. Z. Yan T. M. Xue F. Zhao D. (2013). Rice dry matter and nitrogen accumulation, soil mineral n around root and n leaching, with increasing application rates of fertilizer. Eur. J. Agronomy. 49, 93–103. doi: 10.1016/j.eja.2013.03.008

[B31] Ruidisch M. Bartsch S. Kettering J. Huwe B. Frei S. (2013). The effect of fertilizer best management practices on nitrate leaching in a plastic mulched ridge cultivation system. Agriculture Ecosyst. Environ. 1690, 21–32. doi: 10.1016/j.agee.2013.02.006

[B32] Shcherbak I. Millar N. Robertson G. P. (2014). Global meta analysis of the nonlinear response of soil nitrous oxide (N_2_O) emissions to fertilizer nitrogen. Proc. Natl. Acad. Sci. United States America. 111 (25), 9199–9204. doi: 10.1073/pnas.1322434111 PMC4078848 24927583

[B33] Sun Q. Y. Liu Y. L. Yang H. F. Chen X. Fan X. Sun W. T.. (2022). Applying control-released fertilizer of whole growth duration in seedling-raising pot to effectively decrease the risk of n loss in the paddy field. Plant Nutr. Fertilizer Science. 28 (03), 566–574. doi: 10.11674/zwyf.2021249

[B34] Sun H. F. Zhou S. Zhang J. Zhang X. X. Wang C. (2020). Effects of controlled-release fertilizer on rice grain yield, nitrogen use efficiency, and greenhouse gas emissions in a paddy field with straw incorporation. Field Crops Res. 253, 107814. doi: 10.1016/j.fcr.2020.107814

[B35] Tian C. Zhou X. Huang S. Y. Yuan H. L. Xie G. X. Liu Q.. (2019). Effects of controlled-release urea on CH_4_ and N_2_O emissions and economic benefits in rice fields. Ecol. Environ. Sci. 28 (11), 2223–2230. doi: 10.16258/j.cnki.1674-5906.2019.11.011

[B36] Wang Q. Jiang L. N. Pan J. Q. Ma J. W. Ye J. Zou P. (2019). Nitrogen change and risk assessment of surface water nitrogen loss in paddy field with one-time fertilization. J. Agro-Environment Sci. 38 (1), 168–175. doi: 10.11654/jaes.2018-0204

[B37] Witheetriron Y. Tripathi N. K. Tipdecho T. Parkpian P. (2011). Estimation of the effect of soil texture on nitrate-nitrogen content in groundwater using optical remote sensing. Int. J. Environ. Res. Public Health 8 (8), 3416–3436. doi: 10.3390/ijerph8083416 21909315 PMC3166751

[B38] Xu X. P. He P. Pampolino M. F. Johnston A. M. Qiu S. J. Zhao S. C.. (2014a). Fertilizer recommendation for maize in China based on yield response and agronomic efficiency. Field Crops Res. 157, 27–34. doi: 10.1016/j.fcr.2013.12.013

[B39] Xu X. He P. Qiu S. J. Pampolino M. F. Zhao S. C. Johnston A. M.. (2014b). Estimating a new approach of fertilizer recommendation across small-holder farms in China. Field Crops Res. 163, 10–17. doi: 10.1016/j.fcr.2014.04.014

[B40] Yang Y. C. Tong Z. H. Geng Y. Q. Li Y. C. Zhang M. (2013). Biobased polymer composites derived from corn stover and feather meals as double-coating materials for controlled-release and water-retention urea fertilizers. J. Agric. Food Chem. 61, 8166–8174. doi: 10.1021/jf402519 23923819

[B41] Yang Y. C. Zhang M. Li Y. C. Fan X. H. Geng Y. Q. (2012). Controlled release urea improved nitrogen use efficiency, activities of leaf enzymes, and rice yield. Soil Sci. Soc. America J. 76 (6), 2307–2317. 10.2136/sssaj2012.0173

[B42] Zhang A. P. Liu R. L. Gao J. Zhang Q. W. Xiao J. N. Chen Z.. (2014). Effects of biochar on nitrogen losses and rice yield in anthropogenic-alluvial soil irrigated with yellow river water. J. Agro-Environment Science. 33 (12), 2395–2403. doi: 10.11654/jaes.2014.12.017

[B43] Zhang Y. T. Liu H. B. Wang H. Y. Zhai L. M. Liu S. Lei Q. L.. (2016). A biometric analysis of status and trend of international research on field nitrogen application effects on nitrogen losses and water quality. Acta Ecologica Sinica. 36 (20), 4594-4608. doi: 10.5846/stxb201504140764

[B44] Zhang J. S. Li B. Wang C. Q. Xiang H. Zhou Y. H. Yin B.. (2017). Effects of the blending ratio of controlled release nitrogen fertilizer and urea on soil nitrogen supply in the mid-late growing stage and yield of wheat and rice. J. Plant Nutr. Fertilizer. 23 (1), 110–118. doi: 10.11674/zwyf.16099

[B45] Zhang S. G. Shen T. L. Yang Y. C. LI Y. C. Wan Y. S. Zhang M.. (2018). Controlled-release urea reduced nitrogen leaching and improved nitrogen use efficiency and yield of direct-seeded rice. J. Environ. Management. 220, 191–197. doi: 10.1016/j.jenvman.2018.05.010 29778955

[B46] Zhang A. P. Yang S. Q. Yi J. Yang Z. L. (2010). Analysis on current situation of water pollution and pollutant sources in ningxia yellow river irrigation region. Chin. J. Eco-Agriculture. 18 (6), 1295–1301. doi: 10.3724/SP.J.1011.2010.01295

[B47] Zhang X. Zhang Y. Bi Z. L. Shan Z. X. Ren L. J. Li Q. (2020). Distribution and source analysis of nitrate in surface waters of China. Environ. Science. 41 (04), 1594–1606. doi: 10.13227/j.hjkx.201909078 32608665

[B48] Zhang Y. Zhang X. Bi Z. L. Yu Y. Shi P. Ren L. J.. (2020). The impact of land use changes and erosion process on heavy metal distribution in the hilly area of the loess plateau, China. Sci. Total Environment. 718, 137305. doi: 10.1016/j.scitotenv.2020.137305 32088479

[B49] Zhang X. Zhang Y. Shi P. Bi Z. L. Shan Z. X. Ren L. J. (2021). The deep challenge of nitrate pollution in river water of China. Sci. Total Environment. 770, 144674. doi: 10.1016/J.SCITOTENV.2020.144674 33513508

[B50] Zhang Q. W. Zhang H. Yi J. Luo L. G. Zhang A. P. Wang F.. (2010). The fate of fertilizer derived nitrogen in a rice field in the qingtongxia irrigation area. Acta Sci. Cicumstatiae. 30 (08), 1707–1714. doi: 10.13671/j.hjkxxb.2010.08.023

[B51] Zheng C. Yang Z. C. Qiu X. C. Yin L. Li Y. L. (2018). Analysis on current situation of water pollution and pollutant sources in ningxia yellow river irrigation region. Bull. Soil Water Conserv. 38, 74–79. doi: 10.13961/j.cnki.stbctb.2018.06.012

